# Predictors of pathological gambling behaviours in parents population in Nigeria

**DOI:** 10.1038/s41598-024-56369-8

**Published:** 2024-04-22

**Authors:** Moses Onyemaechi Ede, Kingsley Chinaza Nwosu, Chinedu Ifedi Okeke, Joy Obiageli Oneli

**Affiliations:** 1 Department of Childhood Education, Faculty of Education, University of the Free State, Bloemfontein, South Africa; 2 Division of Student Affairs, University of the Free State, Bloemfontein, South Africa; 3 Teleo Network International School of Theology, Pastoral Ministry Grace Training International Bible Institute, Duluth, Gambia

**Keywords:** Psychology, Health occupations

## Abstract

The increasing incidence of psychological pains, burnout, and anxiety among gamblers in Nigeria is high. This is because pathological gambling (PG) is on the rise and it is linked to many social vices such as stealing, drug abuse, and sexual abuse. It is important to investigate the trajectories of PG in Nigeria. A cross-sectional survey design was employed in our study with 197 participants sampled from 28 gambling venues in Nigeria made up the study’s sample size. Of the 197, 131 (66.5%) were males and 66 (33.5%) were females. 50 (25.4%) were within the age range of 20–30 years, 42 (21.3%) were within 31–40 years, 40 (20.3%) were within 41–50 years, 35 (17.8%) were within 51–60 years, and 30 (15.2%) were within 61 and above. Using the hierarchical regression analysis, our finding revealed a significant association between psychological distress and our respondents’ pathological gambling behaviour whereas none of the sociodemographic variables made a significant contribution to their pathological gambling behaviour. We concluded that gamblers’ psychological well-being is crucial to understanding their problematic gambling behaviours in the context of a developing nation such as Nigeria, and that this could be similar among our respondents. Implications of our findings were highlighted.

## Introduction

Gambling is a legal activity in Nigeria with increased customers patronizing the commercial house, although it seems to lack strict regulatory and enforcement body. This has monumentally increased gambling-related harm, affecting people irrespective of age, class, and location. The National Lottery Regulatory Commission oversees gambling in Nigeria. In Nigeria, the National Lottery Act of 2005 legalized the National Lottery. Lotteries, land-based casinos, and sports betting are examples of legal gambling; roulette, dice games, and low-stakes card games are prohibited. In Nigeria, 18 years is the legal minimum age to be able to gamble^1^.

In Nigeria, gambling is a source of raising tax income or funds for government. The public now views gaming more favorably, particularly among younger people^2^. Online sports betting (such as football league promotions and pools), lotteries, and slot machines are the most widely used types of gambling^3,4^. Others include but are not limited to wagering and betting on the outcome of sporting event or race (football betting, car racing, horse racing, wrestling, basketball, swimming on platforms such as Bet9ja, Naira Bet, Merry Bet, Sure Bet, Sahara Bet, Betpawa Bet, Ebony Bet), virtual games (Bingo, Babynseju, Ajasare), Casino games (kalokalo) and Lottery style games such as Cross-Lotto, Pools (Baba Ijebu), tickets and keno, all of which award prizes based on the selection of winning symbol or number combinations^5^. However, some argue that problem gambling in Nigeria, in the near future, will be a greater public health problem than substance misuse. It is surprising that, despite the nature and scale of this problem, gambling and its related harms have not been adequately researched in Nigeria. Notwithstanding Nigeria’s legal framework, over 57% of school-age population claim having gambled at least once in their lives and of these, 58.3% reported having unrestricted access to gaming establishments^2^.

Pathological gambling was classified as a disorder in 1980 in the DSM-3 edition^6^. Petry et al.^7^ remarked that the name of the disorder was altered in DSM-5 to “gambling disorder”. Pathological gambling disorder also spans problem gambling and compulsive gambling^7^. In this context, pathological gambling disorder is a severe excessive impulse or intention to engage in betting activities that are injurious to oneself and others with making financial or material gains. Petry et al.^7^ noted that “gambling disorder” appears to be the most appropriate name and pathological gambling was included in the Impulse-Control Disorders.

The type of gambling every person engages in may often be dependent upon the gender, age, cultural and ethnic background, and accessibility of the individual^8–11^. However, the forms of gambling that are constant in nature and require an element of skill may have a stronger association with pathological gambling^8,12^. Some studies^8,12^ argued that pathological gambling occurs globally and affects all cultures, ages, genders, and ethnic groups.

Although others posited that pathological gambling is more rampant among youth aged 12–17 years, than the prevalence in the adult population^13,14^. Ahaibwe et al.^15^ revealed that the youth (18–30 years) are more likely to engage in gambling compared to their older counterparts (31 years and above). Ahaibwe et al.^15^ also indicated that participation in gambling is significantly influenced by age, gender, and work status. This can be linked to the fact that today’s youth have the desire to make quick money is another key driver of gambling among the youth. Brezing et al.^8^ noted that estimated adult prevalence of pathological gambling (PG) at 1–2% while estimating the adolescent PG rates at 3–8%. The prevalence of gambling involvement in young adults may also be rising over time as studies estimated increases from 45 to 66% 27–29, and others estimated that young adults gamble frequently ranging up to 91% participation^16^. Shaffer and Hall^17^ found that between 4.4% and 7.4% of young individuals display problematic and pathological gambling behaviours.

Gambling problem cut across ages and even among young, middle and older adults. Verbeke and Dittrick-Nathan^18^ posited that gambling has been long recognized as an adult pastime that is characterized by thrills and risks. In recent years, gambling is growing rapidly among emerging adults, who have grown up in a society where gambling appears to be accepted. A proportion of young adults engage more in gambling activities in a weekly basis and such gamblers exhibit excessive behaviours that are inappropriate^16,17,19^.

The current depressed state of the Nigerian economy appears to have been luring people into ways of making money in the fastest means. The intention to wager money and material things in order to become rich currently seems to be a major concern for most people in Nigeria, probably, because of its negative impacts on people. Apart from the Nigerian context, it has been reported that the proportion of people who engage in gambling is over 760 million world population in 2011 and the majority are above 60 years old^20^. The World Economic Forum projected that the number will increase to 22% (2 billion) by 2050. The widespread of this phenomenon has been traced to several factors. Among the factors are age^9,10^ culture^11^ psychiatric and physical comorbidity^10^. Even though there may be monetary gain attached to gambling or betting it could expose emerging adults to some frequent disciplinary problems and maladaptive behaviours in society. They bet increasingly to cover up past losses to the extent that they have lost control and become addicted to betting. Some find it difficult to concentrate on schoolwork and obey family rules and regulations.

Relating pathological gambling to the social development of children and the notion of social learning theory, this study considered the attention, retention, and motivation of parents with involvement in gambling activities to influence the social and academic development of their children. The social learning perspective, therefore, has possible implications for early childhood development. The Bandura social learning theory places a strong emphasis on observing and imitating the actions, attitudes, and feelings of others. According to Bandura^21^, learning would be extremely difficult and maybe dangerous if people were forced to only rely on the results of their own activities to guide them in making decisions. Fortunately, the majority of human behavior is acquired by modeling and observation: through watching others, one develops an understanding of how new behaviors are carried out, and this coded information later on acts as a guide for action. According to the social learning views, environmental, behavioral, and cognitive forces constantly interact to shape human behavior.

### Problem and motivation

The present rates of gambling activities are becoming alarming and bemoaning. Some people who gamble tend to develop health-related problems such as an increased risk of alcohol and substance abuse disorders^22^, high risk of suicide impulses, higher anxiety^16^ and poor general health^23^. Studies have revealed that tense relationships, delinquency, illegal behaviour, depression, and even suicide are problems gamblers face in society^24,25^. In addition, some of them suffer from physical disabilities and some health problems^26^. Worst still, studies indicated that between 4 and 8% of gamblers have tense gambling problems, while another 10–15% are at-risk^25,27,28^.

In the Nigerian case, gamblers appear to be engaging in inappropriate behaviours such as fighting, stealing, weapon carrying, heavy alcohol consumption, illicit drug abuse, and destruction of public properties each time they lose valued material things^3,29,30^. The researchers’ observation tends to show that young, middle, and older adults exhibit disruptive gambling behaviour that impacts their interpersonal relationships with their family and friends. Experiences have revealed that young adults unceasingly surf the internet in order to gamble against academic or vocational pursuits^30^. It seems worrisome as some of them who are pathological gamblers are associated with significant impairment and distress^31^, traditional substance-related addictions, salience, mood modification, abandonment, conflict, and deterioration^32^. This causes negative or unhelpful impacts on their careers^33^. Such negative outcomes have short- and long-term implications for the individual, parents, guardians, caregivers, as well as society at large^34^.

It also implies that if the male and female expectancies become negative, the students may experience negative emotional consequences and loss of control, especially frequent gamblers. So, it may be inferred that differential negative emotional consequences and loss of control are significantly associated with gambling frequency. Meanwhile, Gbadebo^35^ noted that students in Nigeria, Southeast Inclusive, get hooked on internet activities such as pornography, internet gaming, video gaming, online shopping, internet theft, searching for non-important information, or chatting for a very long time. With respect to the activities available on the internet, Loh et al.^36^ remarked that a variety of related activities are available on the internet.

Kuss^31^ called for research that would be focused on problematic gambling behaviours and there is a need for researchers to conduct research on this since the existing studies seemed not to be enough. Also, population-based empirical study of pathological gambling participation is limited^37^, especially in southeast Nigeria^3,30^. Another study reported that information on gambling disorder (GD) in Nigeria is lacking, a nation that has the second-biggest market for online sports gaming in Africa^38^. It is decrying that despite the associated hazards attributed to pathological gambling, there are few studies on pathological gambling in Nigeria and it is unexpected given the scope and nature of the issue^1^. Calling for critical stakeholders (academics, healthcare professionals, and policymakers) to engage in positive action that will ensure information dissemination, outreach programme, and research reports minimising gambling-related harm to the people of Nigeria^1^. The researchers are uncertain if pathological gambling disorder is more prevalent in Nigeria or whether pathological gambling disorder is dependent on age, location, or gender. It is, therefore, in view of this background that this research aimed at ascertaining the degree to which participants’ sociodemographic variables and psychological distress predict pathological gambling. The following hypotheses guided this study:

- Psychological distress will be a significant predictor of pathological gambling.
- Socioeconomic status and employment status will be significant predictors of pathological gambling.
- Number of Children and marital status will be significant predictors of pathological gambling.
- Age, gender, and location will be significant predictors of pathological gambling.
- Socioeconomic status and employment status will account for a significant amount of variance above and beyond psychological distress scores.

## Methods

### Design of the study

A cross-sectional survey research design was adopted in the study. It shows the strength of the relationship between two or more characteristics of subjects^39^. This design was used because the researchers ascertained if sociodemographic and psychological variables predict pathological gambling disorder among adults in Nigeria. The study was carried out in Ebonyi and the Enugu States of Nigeria. Ebonyi and Enugu States are in the South-Eastern part of Nigeria.

### Participants

The participants of this study were selected from different gambling venues in Enugu state Nigeria. A total of 197 people were signed up to participate in the study. The researchers with help of research assistants adopted an accident sampling technique which helped the researcher to collect 197 comprising young, middle, and older adults. Also, there are male and female, student gamblers and out-of-school gamblers, some are married while others are not with different affiliations (e.g., religion, ethnicity) as well as different academic qualifications. More details about the socio-demographic characteristics of the participants can be found in Table 1. Three 28 betting centres were purposively selected especially where the researchers met people who engaged in gambling. By doing this, some criteria were applied while choosing bet shops. These include the shops must be located either in Ebonyi or Enugu State, must have a record book for record-keeping, and must operate according to the National Lottery Act 2005. To ensure that the number of participants was statistically accurate and adequate enough as suggested by Faul^40^. Faul’s Gpower statistical tool confirmed the power of the sample size.

**Table 1:** Demographic characteristics of the study participants (n = 197).

Characteristics	Category	Frequency	Percent
Gender	Male	131	66.5
	Female	66	33.5
Age	20–30 years	50	25.4
	31–40 years	42	21.3
	41–50	40	20.3
	51–60 years	35	17.8
	61 and above	30	15.2
Social economic status	Low	86	43.7
	Moderate	78	39.6
	High	33	16.8
Number of children	Below six	112	56.9
	Six and above	85	43.1
Location	Urban	65	33.0
	Rural	132	67.0
Marital status	Single	17	8.6
	Married	100	50.8
	Divorced	44	22.3
	Separated	36	18.3
Employment status	Employed	73	37.1
	Unemployed	124	62.9

Table 1 shows the socio-demographic information of participants. It shows that 131 (66.5%) male and 66 (33.5%) female respondents responded to the measures. Of all the total number, 50 (25.4%) were within the age range of 20–30 years, 42 (21.3%) were within 31–40 years, 40 (20.3%) were within 41–50 years, 35 (17.8%) were within 51–60 years, and 30 (15.2%) were within 61 and above. 86 (43.7%) respondents had low social economic status, 78 (39.6%) had a moderate social economic status, and 33 (16.8%) of them had high social economic status. In terms of the number of children they have, 112 (56.9) respondents have below six children, 85 (43.1%) of them have six and above. Regarding the location, 65 (33.0%) respondents were from urban and 132 (67.0%) were from rural locations. From their responses, 17 (8.6%) were single, 100 (50.8) were married, 44 (22.3%) were divorced, and 36 (18.3%) were been separated from their marriages. Regarding the respondents’ employment status, 73 (37.1%) were employed while 124 (62.9%) were not employed.

### Ethical compliance and procedure

This study was retrospectively registered in the UMIN clinical registry with the Unique ID: UMIN000051521. This work has been performed in accordance with the Declaration of Helsinki. University of Nigeria’s ethical committee under the auspices of the Faculty of Education approved that the researcher should conduct this study. In addition to that, a permission letter was obtained from administrative authorities before the conduct of the study. At the point of reaching each gambling centre, participants were solicited to sign up for the study if they wished to participate. Informed consent was obtained from all subjects and/or their legal guardian(s). Thereafter, an envelope that contained copies of questionnaires, pen, and pencil. All in all, all methods were performed in accordance with the relevant guidelines and regulations by including a statement in the methods.

All participants were asked to move to venues designated by the researchers called collection centres. The collection centres are places identified by the researchers and very close to each gambling venue. Each participant was given a packet of an envelope to complete the instruments within 30 to 40 min. The sitting arrangement during the exercise was an average 7 m away from one participant to another. They were given the opportunity to ask a question if there was any item in the instrument that posed a challenge or they did not understand. There was no incentive given during the study. About 2% of the gamblers who assented to participate in the study returned the instrument uncompleted.

### Measures

Kessler’s Psychological Distress Scale (K10) assessed the frequency of depressive and anxiety symptoms over the past month on a 4-point Likert scale^41^. The scale has 10 items. This tool has been validated to screen for common mental disorders in developing country settings including India^42,43^. The scale is rated in a 4-Likert option that ranges from None of the time = 1 to All of the time = 5. Any parent with a score of less than 20 is likely to be healthy; those with a score of 20–24 are likely to have a mild mental problem; those with a score of 25–29 are likely to have a moderate mental disorder, and those with a score of 30 or more are likely to have serious mental symptoms. A quarter of the adult population will have a score of 20 or higher, and one out of every four patients seen in primary care will have a score of 20 or higher. In this study, the internal consistency of Kessler’s Psychological Distress Scale was 0.72.

The PGSI (Problem Gambling Severity Index) is made up of nine items that are assessed on a four-point scale (0 = Never, 3 = Almost Always) and are based on prior year experiences^44^. The researchers utilized a PGSI 5 criterion for problem gambling (which has been demonstrated to have the highest classification accuracy when compared to clinician evaluations including thorough case conceptualizations), with PGSI 1–4 indicating low to moderate severity issues (given that all such responders were demonstrating at least some signs of problematic gambling). We found a high reliability (0.78) of PGSI.

Participants Demographic Inventory (PDI) is a checklist that finds out the personal information of the participants such as age, gender, socioeconomic status, residence, and working status.

### Data analysis

The data collected was analyzed using correlation and hierarchical multiple regression statistics. Correlation analysis was used to test the relationship between the dependent variable and the predictor variables. The hierarchical multiple regression was employed to test the prediction of pathological gambling, by psychological distress and, controlling for socioeconomic status, employment status, number of children, marital status, gender, age, and location.

## Results

Table 2 shows the correlation analysis of the dependent variable and the predictor variables. The result shows that psychological distress has a significant positive relationship with pathological gambling (r = 0.483, p ˂ 0.001). This demonstrates that there is a positive association—that is, that psychological distress is a predictor of PG. On the other hand, Socioeconomic status (r = 0.050, p ˃ 0.244) and the number of children (r = 0.032, p ˃ 0.326) are positively related to pathological gambling. For employment status (r =  − 0.020, p ˃ 0.388), marital status (r =  − 0.078, p ˃ 0.138), gender (r =  − 0.120, p ˃ 0.047), age (r =  − 0.021, p ˃ 0.383), and location (r =  − 0.014, p ˃ 0.425) were not significantly related to pathological gambling. This implies that persons with psychological distress have higher tendencies for pathological gambling while persons without psychological distress symptoms have lower tendencies for pathological gambling.

**Table 2:** Correlation analysis of the dependent variable and the predictor variables (*N* = 197).

S/N	Variables	1	2	3	4	5	6	7	8	9
1	PGS	–	− 0.120*	− 0.021	0.050	0.032	− 0.014	− 0.078	− 0.020	0.483**
2	Gender		–	0.190**	− 0.092	− 0.054	− 0.119	0.119*	− 0.012	− 0.123*
3	Age			–	− 0.133**	− 0.086	0.019	0.011	− 0.018	− 0.133**
4	SE status				–	0.068	− 0.081	0.185**	0.092	0.095
5	No of Children					–	− 0.064	0.061	0.138**	0.149**
6	Location						–	− 0.004	− 0.114	− 0.012
7	Marital Status							–	− 0.063	− 0.053
8	Employment Status								–	− 0.079
9	KPDS									–
–	Mean									27.97
–	SD									1.94

*PGS* problem gambling severity, *KPDS* Kessler’s psychological distress scale, *SES* socioeconomic status, *ES* employment status, *NC* number of children, *MS* marital status.

**Correlation is significant at the 0.01 level (2-tailed).

*Correlation is significant at the 0.05 level (2-tailed).

Table 3 shows the hierarchical regression analysis that was conducted to predict our respondents’ pathological gambling behaviours. The predictor variables employed in the regression analysis were entered in three stepwise manner. In the first step, psychological distress was entered as the possible predictor variable of pathological gambling behaviour. The result revealed that PG, *R*^2^ = 0.234, *F* (1,195) = 59.433, *p* ˂ 001)*, R*^2^ = 0.234. In the second step, the predictor variables economic status and employment status were added to the model which resulted in the model not accounting for any changes in the criterion variable. However, there was no joint significant association between the predictor variables in this second step and the criterion variable *F* (2, 193) = 19.643, *p* ˂ 0.960), *R*^2^ = 0.234, ∆*R*^2^ = 0.000. Looking at the individual contributions, neither economic status nor employment status *β* = 0.483, *t* (7.709) = 0.975, and *β* = 0.018, *t* (0.280) = 0.780 respectively had any significant contribution to the variances in our respondents’ pathological gambling behaviours.

**Table 3:** Hierarchical multiple regression predicting pathological gambling, by psychological distress and, controlling for socioeconomic status, employment status, number of children, marital status, gender, age, and location.

Model		*B SE B*		*Β*	*T*	*Sig.*	*R*	*R*^*2*^	*∆R*^*2*^	*∆F*	*P* = ∆*F*
1	KPDS	0.285	0.037	0.483	7.709	0.000	0.483	0.234	0.234	59.433	0.001
2	KPDS	0.286	0.038	0.485	7.626	0.000	0.484	0.234	0.000	0.041	0.960
	Socioeconomic status	0.005	0.169	0.002	0.032	0.975					
	Employment status	0.071	0.254	0.018	0.280	0.780					
3	KPDS	0.287	0.038	0.487	7.518	0.000	0.488	0.238	0.004	0.546	0.580
	Socioeconomic status	0.037	0.173	0.014	0.213	0.831					
	Employment status	0.077	0.259	0.019	0.298	0.766					
	Number of children	− 0.160	0.253	− 0.041	− 0.630	0.529					
	Marital status	− 0.111	0.141	− 0.051	− 0.784	0.434					
4	KPDS	0.287	0.039	0.486	7.407	0.000	0.494	0.244	0.006	0.513	0.674
	Socioeconomic status	0.033	0.176	0.012	0.187	0.852					
	Employment status	0.073	0.261	0.018	0.278	0.781					
	Number of children	− 0.160	0.255	− 0.041	− 0.627	0.532					
	Marital status	− 0.094	0.143	− 0.043	− 0.658	0.512					
	Gender	− 0.279	0.271	− 0.068	− 1.029	0.305					
	Age	0.077	0.091	0.055	0.846	0.399					
	Location	− 0.068	0.266	− 0.017	− 0.257	0.797					

*B* Unstandardized regression coefficient, *β* Standardised regression coefficient, *∆R*^*2*^ Change in *R*^2^, *∆F* Change in F, *PGS* Problem Gambling Severity, *KPDS* Kessler’s Psychological Distress Scale.

In the third step, number of children and marital status were entered into the model. There was also no joint significant contribution to the variances in the responses on pathological gambling behaviours, accounting for 23.8% variances in the respondents’ PG, that is, an additional 0.04% above models 1 and 2, *R*^2^ = 0.238, ∆*R*^2^ = 0.004; *F* (2, 191) = 11.949, *p* ˂ 0.580, these additional predictor variables made no significant individual contributions to the model *β* = 0.483, *t* (7.709) = 0.831; *β* = 0.014, *t* (0.213) = 0.780 respectively. Model 4 shows that psychological distress, employment status, socioeconomic status, number of children, marital status, location, age, and gender when taken together did not significantly account for additional variance above and beyond psychological distress scores, *R*^*2*^ = 0.244, ∆*R*^2^ = 0.006; *F* (3, 188) = 7.603, *p*˂0.674; and there were no significant individual contributions to the variances in the PG of our respondents by our respondents’ location, age and gender.

## Discussion

Our study investigated factors that predict pathological gambling behaviours among parents in Nigeria using hierarchical regression analysis. We found that pathological gambling behaviours was significantly predicted by psychological distress whereas the addition of socio-demographic variables in the models, made no significant variances in the pathological gambling behaviours among Nigerian parents. Individually, none of the socio-demographic variables was able to make a significant contribution to the variances in our respondents’ pathological gambling behaviours.

Our finding that showed a significant relationship between psychological distress and pathological gambling behaviours of our respondents is consistent with the existing body of literature that has demonstrated that psychological distress is associated with gambling^45,46^. The relationship between psychological distress and our respondents’ gambling problems was so strong that even when the socio-demographic variables were added to the model, the association remained strong. It indicated that the higher the psychological distress of our respondents, the more likely they are vulnerable to engage in problematic gambling behaviours. Similar studies have revealed that people who have problematic gambling behaviours are more likely to have higher psychological distress scores than non-gamblers^36^. There have been attempts to unravel the link between psychological distress and pathological gambling behaviours. A study conducted among Finish and US respondents has shown that irrespective of the fact that psychological distress has a direct effect on gambling behaviours, consumer debt, and debt problems could be a factor that can explain the association between these two variables especially among Finish respondents^47^. This explains that when gamblers face gambling problems they are likely to have deteriorating well-being. Similarly, laboratory gambling experiments have demonstrated that sensitivity to stress may predispose people to high-risk gambling behaviours^48^. Indicating that people who may be distressed are likely to experience gambling problems.

Furthermore, the present results revealed no significant joint and individual variable contributions of the economic and employment status variable to variances in our respondents’ pathological gambling behaviours scores in the second model two. What this implies is that a unit increase in our respondents’ economic and employment status did not have a consequent increase or decrease in their pathological gambling behaviours. This contradicts the findings of some related studies that showed that unemployed people are less likely to engage in gambling than those who are employed^15^. The researchers explained that even though the unemployed may engage in gambling they are limited to gambling engagement due to financial constraints. Similarly, a perceived economic status which encompasses the way and manner they perceived their earnings and occupational prestige among themselves did not also account for the variances in their pathological behaviours. This also contradicts similar findings that show that lower economic status is linked to problematic gambling. In our study, there is no association between gambling behaviours and economic status. However, there are indications that low economic status could predispose people to at-risk gambling^49^.

More so, the third model did not show any significant improvement to the model. The *∆R*^*2*^ was insignificant. Whereas the number of children and marital status made no individual contributions to the variances in the respondents’ pathological gambling behaviours, their psychological distress remained a significant predictor of the outcome variable. Studies that focused on marital status and gambling have revealed that being un-partnered associated with loneliness could result in gambling problems, and that un-partnered older adults are likely to have gambling problems^50^. Reasons advanced for this is that they are likely to be lonely resulting in their continuous engagement in gambling. Our finding did not show that marital status accounted for a variation in our respondents’ gambling behaviours, thereby contradicting this view. Our results also showed that the number of children our respondents have is not associated with pathological gambling behaviours. Though, there appears to be no evidence in literature on how the number of children in a household is associated with parents’ gambling behave, it could be theorized that financial stress associated with having higher number of children could be associated with less attempts to engage in gambling. Hence, expectations could be that those who have more children are less likely to have more gambling problems than those who do not few children. This could be supported by the fact that studies have shown that gambling is associated with higher household income^51^. However, our findings contradicted this assumption.

Our last model did not indicate a significant contribution of the addition of gender, age, and location (primary residence of the respondents) to the model. There was also no significant joint contribution to the variances in our respondents’ pathological gambling behaviours, the *∆R*^2^ remained insignificant. Gender was not a significant predictor of pathological gambling behaviours in our study. A plethora of studies have shown how significant gender is in gambling research. Venne et al.^52^ have noted that gambling has been regarded as a gender-based activity. Castrén et al.^53^ found that gender is significantly associated with gambling expenditure, and women are less likely to engage in betting than men^54^. Being male could be a risk factor in harmful gambling^55^, given that males are more inclined to strategy games than their female counterparts. However, our findings showed a non-significant association of gender with problematic gambling behaviours implying that problematic gambling behaviours may be similar across gender among our respondents. This aligns with a recent understanding that has shown that, in many respects, women and men may have similar gambling experiences^52^. Similarly, age was not a significant predictor of gambling behaviours among our respondents. This implies that variances in pathological gambling behaviours among our respondents were not dependent on the age bracket they fall in. Our findings appear to contradict similar findings that have indicated that age is a significant factor in gambling behaviours problems^56^, and that older gamblers are likely to have fewer gambling problems than younger gamblers^10^. In our study we considered the residence of our respondents as being an important factor in predicting their gambling behaviours. However, our findings indicated no significant variances accounted for by the primary residence of our respondents implying that where our respondents live may not be a significant factor in determining whether they will have pathological gambling behaviours or not. Previous studies are indicative that urbanity has negative effects on gambling expenditure^51^, and gambling problems are estimated to be higher among rural dwellers than urban dwellers^57^.

## Conclusions, implications, and limitations

Our findings have demonstrated the complementary relationship between psychological distress and the pathological gambling behaviours of our respondents while controlling for their demographic variables using a multiple-stepwise regression model. The impact of psychological distress remained significant on our respondents’ pathological gambling behaviours irrespective of the inclusion of demographic variables in the models. We conclude from the findings that gamblers’ psychological well-being is crucial to understanding their problematic gambling behaviours in the context of a developing nation such as Nigeria and that this could be similar among our respondents.

There are significant theoretical and practical implications regarding the significant impact of psychological distress on our respondents’ pathological gambling behaviours. First, it could be that problematic gambling behaviours are follow-up to the distresses gamblers may encounter as a result of losses they encounter during gambling endeavours. Within the Nigerian context where it has been noted that the quest for quick money and avarice may be responsible for a positive attitude to gambling^58^, the incongruity that may exist between this quest and the reality could lead to distress which is likely to lead to pathological gambling behaviours given that psychological distress has been linked to cognitive distortion^59^, and pathological gambling associated with biased information^60^. Practical implications of our study include the fact that for problematic gambling behaviours to be addressed there is a need that interventions programmes targeted at enhancing their psychological well-being are mounted. This could be in the form of cognitive-based psychotherapies.

As the therapists assist the problem gamblers, there should policy framework that will recognise the percentage of males and females who have mental health issues. The recognition would give room for the provision of mental health services irrespective of their locations. Hopefully, this will offer a practical approach to gambling operatives. By extension, the gambling operation will also receive regulatory structure expanding to divergent populations. For instance, creating algorithms to identify risky play levels (in online environments) or by voluntarily imposing time and financial constraints^61^. Following the above premises, scientific society such as gambling researchers and mental health researchers could set up investigative research to examine the achievement and progress. This is because there are need for a systematic reframing of the psychological and health problems that are associated with pathological gambling and how they affect communities, individuals, and families^62,63^. The engagement of scientific communities and policymakers will strengthen the efforts to reduce the negative influence of commercial, governmental, and regulatory forces in forming the conditions that give rise to these harms.

Notwithstanding that our study has made a significant contribution to gambling studies—being the first study within the Nigerian context to examine the impact of psychological distress on gambling behaviours while controlling for socio-demographic variables—it has some limitations that may compound its generalizations. First, reliance on respondents’ responses without observation of their actual gambling behaviour would not warrant that causal relationships among the variables under examination are drawn. However, the nature of the study makes this design appropriate. One should be careful to interpret the findings in the light of the cause-and-effect paradigm. Second, the use of only questionnaire for data collection may likely be a limitation to the generalization of our study. Future research may need to use a mixed-method approach so that findings will be triangulated.

## Data Availability

The datasets used and/or analysed during the current study are available from the corresponding author on reasonable request.

## Abbreviations

PGS: Problem gambling severity
KPDS: Kessler’s psychological distress scale
SES: Socioeconomic status
ES: Employment status
NC: Number of children
MS: Marital status
SD: Standard deviation
*B*: Unstandardized regression coefficient
β: Standardised regression coefficient
*p-value: Significance
∆*R*^2^: Change in *R*^2^
∆*F*: Change in F
